# Structure diagnostics of heterostructures and multi-layered systems by X-ray multiple diffraction^1^

**DOI:** 10.1107/S1600576717006574

**Published:** 2017-05-25

**Authors:** Mariana Borcha, Igor Fodchuk, Mykola Solodkyi, Marina Baidakova

**Affiliations:** a Solid State Physics Department, Yuriy Fedkovych Chernivtsi National University, Kotsyubynskiy Street 2, Chernivtsi, 58018, Ukraine; b Department of Modern Functional Materials, ITMO University, 49 Kronverkskiy Prospekt, St Petersburg, 197101, Russian Federation

**Keywords:** X-ray multi-beam diffraction, lattice parameters, strain, multi-layered systems

## Abstract

This article presents the results of research on multi-layered heterostructures by a modified calculation technique of multiple X-ray diffraction. The Al*_x_*In_1−*x*_Sb heterostructure and a Zn(Mn)Se/GaAs(001) multi-layered system were used as models to specify conditions for cases of coincidental coplanar three-beam or coincidental noncoplanar four-beam X-ray diffraction. These conditions provide the means for a high-precision determination of lattice parameters and strain anisotropy in layers.

## Abstract 2

Opportunities of multi-beam X-ray diffraction are demonstrated for determination of lattice strains in heterostructures and multi-layered systems.

## 1. Introduction   

Research on the internal strain distribution, the composition and the nature of multi-component phases and multi-layered systems, the laws of deposition from solid solutions, determination of phase boundary localization, and measurement of thermal expansion coefficients are only some of the traditional directions of X-ray diffraction application. A wealth of information has been accumulated to date about the methods of determination of lattice parameters of crystalline materials, as well as the techniques and procedures for improving their accuracy. The accuracy of lattice parameter determination is generally limited by the precision of the used wavelength (radiation line width and divergence) in most X-ray diffraction methods, and usually 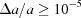. At the same time the Bond method and the method of LLL interferometry (Burke & Tomkeieff, 1969 ▸; Berger, 1984 ▸, 1986 ▸; Härtwig *et al.*, 1994 ▸; Becker, 2001 ▸) give a much greater accuracy, at the level 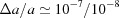.

Multi-beam X-ray diffraction (MBXRD), based on the multi-beam ‘*Umweganregung*’ effect (Renninger, 1937 ▸; Chang, 1984 ▸), is a high-precision instrument for the determination of lattice parameters (Chang, 1984 ▸; Kohn, 1991 ▸; Härtwig *et al.*, 1994 ▸) and stress distribution in complex crystalline systems and materials (Hayashi *et al.*, 1999 ▸; Chang, 2001 ▸; Morelhão *et al.*, 2011 ▸). At the same time, MBXRD is rarely used for research into complex multi-layered semiconductor systems owing to the complexity of the experimental scheme and the difficulty of selecting diffraction planes appropriate for inducing special cases of multi-beam diffraction.

A special feature in lattice parameter determination by the multi-beam technique is the measurement of relative angular positions of multi-beam reflections, instead of absolute angular position of single reflections. Through a combination of multi-beam effects and some intentional influence that changes the lattice parameters (for instance, heating or cooling), this technique can give minimal errors related to instrumental factors, resulting in 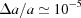 taking into account all possible errors (Kshevetsky *et al.*, 1985 ▸; Borcha *et al.*, 2009 ▸).

This article describes a hypothetical study of multi-layered heterostructures to exemplify a calculation procedure of MBXRD for determination of lattice parameters and strain in each layer of such systems. We implement the possibility to choose the appropriate conditions for each individual heterostructure layer in order to realize unique cases of multi-beam X-ray diffraction, in particular, coincidental coplanar three-beam or noncoplanar four-beam diffraction.

## 2. Features of multi-beam X-ray diffraction   

Renninger (azimuthal) scanning is carried out by rotating the crystal around the diffraction vector of a primary reflection (usually forbidden or quasi-forbidden by the structure) (Chang, 1984 ▸). As a result, the diffraction conditions are sequentially satisfied for other planes and a typical Renninger diffraction pattern consists of a system of multi-beam maxima (reflections). It contains information from many different crystallographic directions, from which the unit-cell parameters can be determined with good accuracy. Each involved crystallographic plane gives two peaks of intensity (when the site of the reciprocal lattice goes into and comes out of the Ewald sphere). Of course, selection of the primary reflection and wavelength is important here, because it affects the accuracy of the determination of the peaks’ angular positions and the shape of the intensity distribution. Lattice parameters are found from the experimentally measured angular distances Δφ between corresponding maxima of multi-beam diffraction (reflections) at a known X-ray wavelength λ (Chang, 1984 ▸). A primary reflection forbidden by the structure is preferred because it ensures minimal background intensity. This method gives the possibility of minimizing errors related to absorption, sample displacement, inaccuracy in the angular positions measured on the detector and other systematic errors inherent in most methods of lattice parameter determination (Chang, 1984 ▸; Kshevetsky *et al.*, 1985 ▸; Härtwig *et al.*, 1994 ▸). For instance, a diffraction pattern has a symmetry that depends on the lattice symmetry. A misalignment of the sample can cause a difference between identical (symmetric) parts of a multi-beam X-ray diffraction pattern. This difference is used to correct the experimental data and to determine systematic errors. This correction is analogous to 180° turning in the Bond method. Thus the MBXRD method is as accurate as the most accurate two-beam methods of lattice parameter measurement, in particular the Bond method.

Rossmanith *et al.* (2001 ▸) developed an algorithm and corresponding software, based on the kinematical theory of X-ray scattering, for qualitative and quantitative analysis of multi-beam reflection in the case of Bragg diffraction. The purpose of this software was the calculation and graphic representation of the intensity distribution of multi-beam reflection during φ scanning of a crystal around the diffraction vector of the primary reflection. Combining a similar approach and the approach suggested by Kshevetsky *et al.* (1985 ▸), it was shown (Borcha *et al.*, 2009 ▸) that the accuracy of this method can be increased, if the cases of coplanar three-beam or noncoplanar four-beam diffraction are implemented. These cases occur when the angular positions of two multi-beam reflections coincide with a certain 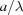 ratio. A change in lattice parameter or wavelength can satisfy the conditions of coincidental diffraction, owing to which reflections can converge and finally superimpose: coincidental coplanar diffraction corresponds to superposition of reflections with the same indexes and coincidental noncoplanar diffraction to reflections with different indexes. For instance, in the work of Borcha *et al.* (2009 ▸) coincidental multi-beam diffraction was achieved by a change of *a* in the process of sample heating at a fixed wavelength. As long as there is no need to measure the angular distance between multi-beam intensity maxima, an opportunity to increase the accuracy of measurement of *a* values arises.

The accuracy of measurement of the angular distance between reflections Δφ (in addition to the accuracy of λ) makes the main contribution to the error of lattice parameter determination. The systematic errors in the determination of the position of each peak are the same (because it is the same scan under the same experimental conditions).

The dependence of the accuracy of lattice parameter determination on the accuracy Δφ of the angular distance φ_*ij*_ between reflections *i* and *j* (for cubic crystals) is given by
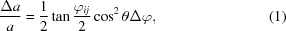
where θ is the Bragg angle of the primary reflection. The special cases of diffraction such as coincidental coplanar or noncoplanar diffraction, when φ*_ij_* = 0, should be used to reduce this error.

## 3. Application of MBXRD calculation procedure   

To investigate the intensity distribution in the region of multi-beam Laue diffraction in a Ge crystal with (depth-dependent) one-dimensional strain, we previously used (Borcha *et al.*, 2005 ▸) an algorithm based on the solution of Takagi’s equations (Takagi, 1962 ▸). This algorithm was modified for the case of Bragg diffraction. This allowed us to take into account the possible influence of one-dimensional depth distortion on the position and shape distributions of X-ray intensity in multi-beam reflection. In contrast to the cubic single crystal, lattice parameters in heterostructures and multi-layered systems can be different in different crystallographic directions (Ashwin *et al.*, 2013 ▸). In such cases, the tetragonal deformations are well defined. As an example of multi-beam diffraction application we have calculated fragments of multi-beam scans for heterostructures and wide-gap multi-layered II–VI compounds that are promising for the manufacture of optoelectronic devices in the green and green–yellow spectral range (Sorokin *et al.*, 2015 ▸).

Several issues arising in this research should be mentioned:

(1) We use the kinematic approach of X-ray scattering because the layer thickness is smaller than the extinction depth. However, taking into account the dynamical effects in future work will give the possibility to determine the strain distribution more precisely, for example, as is done by Larsen *et al.* (2005 ▸).

(2) We researched the angular displacements of multi-beam reflections in epitaxial systems in contrast to other applications of multiple diffraction, where the shape and intensity distribution in multi-beam reflections were analysed (Morelhão *et al.*, 2002 ▸, 2011 ▸; Kyutt & Scheglov, 2013 ▸).

(3) We suggest the experimental conditions (primary reflection and X-ray wavelength) at which multi-beam diffraction will occur only from the studied layer or substrate. But there are some cases when multi-beam X-ray diffraction is implemented simultaneously in the layer and the substrate (Morelhão *et al.*, 2002 ▸).

(4) A whole (360°) Renninger scan has a number of systems of multiple structurally equivalent peaks corresponding to different crystallographic planes. In addition, multi-beam diffraction, in contrast to the two-beam case, gives an opportunity to determine the phases between the interacting waves (Chang, 1984 ▸). At the same time, complex methods involving rocking curves (Morelhão *et al.*, 2005 ▸) and reciprocal space maps, obtained using Renninger scanning or the 2θ–φ scanning technique (Domagała *et al.*, 2016 ▸), would provide reliability and remove the ambiguity in identifying the causes of changes in lattice parameters in heterostructures.

(5) The use of synchrotron radiation allows one to choose an X-ray wavelength that creates conditions for coincidental coplanar three-beam or noncoplanar four-beam diffraction.

We here investigate the possibility of realizing coincidental diffraction in layered systems and show how it can be used for the diagnostics of strain (lattice parameter changes) in the layers. The theoretical sensitivity of multi-beam diffraction to strain and composition of epitaxial layers is analysed.

### 3.1. Al*_x_*In_1−*x*_Sb heterostructure   

Heterostructures with Al*_x_*In_1−*x*_Sb layers are used for quantum well formation at *x* ≤ 0.16. In the process of their creation, the control of Al content is extremely important. For this system *a* varies linearly with Al content in a layer and is determined by the Vegard law. However, the energy gap *E*_g_(*x*) varies nonlinearly (Komkov *et al.*, 2011 ▸): 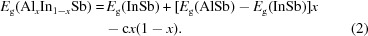
Implementation of three-beam coplanar or four-beam noncoplanar X-ray diffraction in Al*_x_*In_1−*x*_Sb layers can be related to *x* and geometrical diffraction conditions.

**Figure 1:**
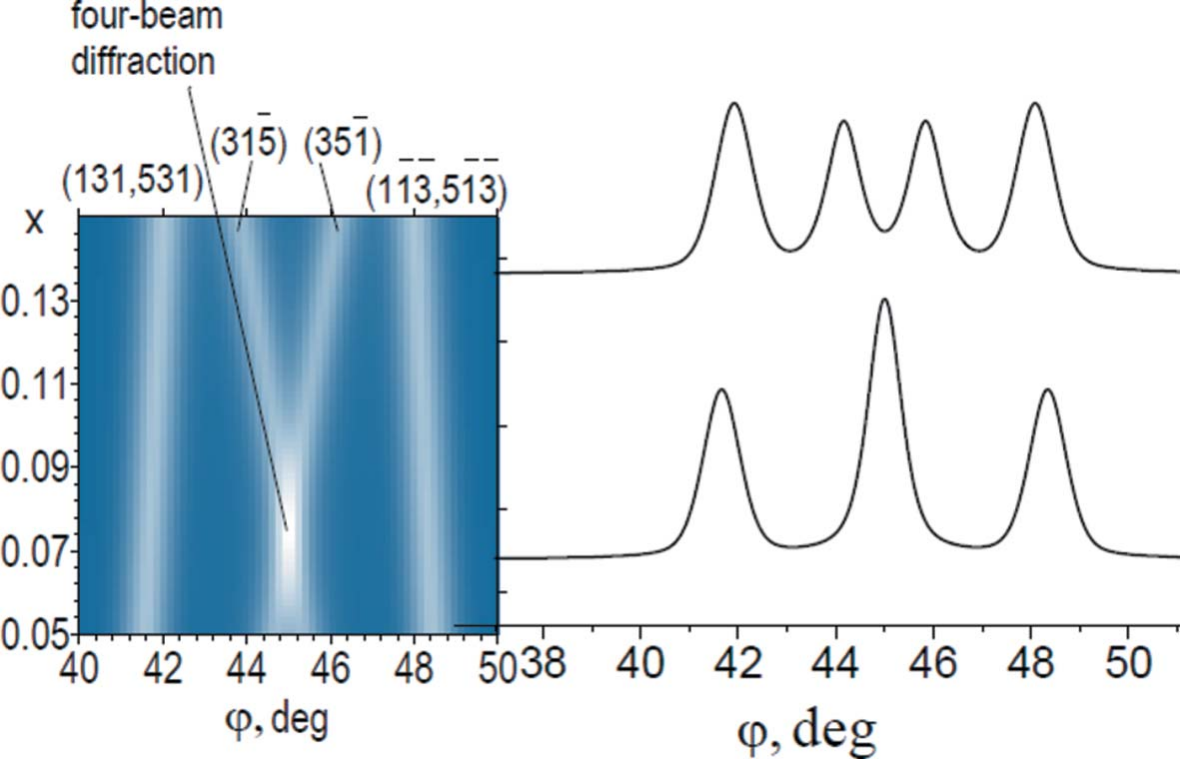
Pattern composed of calculated fragments of diffraction scans with three-beam 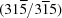 and 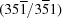 reflections for Al*_x_*In_1−*x*_Sb.

The simulation of fragments of multi-beam scans shows that the most suitable configurations for implementation of four-beam noncoplanar diffraction are three-beam 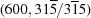 and 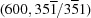 reflections for the primary (600) reflection of Co *K*α_1_ radiation (λ = 1.78611 Å). This is because they are structurally and spectrally sensitive and meet the condition Δφ*_ij_* → 0 (where Δφ*_ij_* is the angular distance between maxima *i* and *j*) (Borcha *et al.*, 2009 ▸). The whole set of calculated diffraction scans is given in Fig. 1 ▸, which demonstrates the dynamics of the 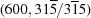 and 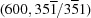 reflections, *i.e.* changes of their angular positions *versus* *x* and overlap at *x* = 0.075, with implementation of three-beam coplanar diffraction.

The superposition of these reflections at *x* = 7.5% leads to minimization of instrumental errors in the lattice parameter determination. From the geometry of the experiment, 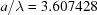. Then we obtain *a* = 6.45358 Å, *i.e.* the accuracy of *a* is determined by λ. Using the capabilities of the spectral range of synchrotron radiation facilitates satisfaction of the condition of coplanar three-beam diffraction for arbitrary *x*. Specifically, for *x* = 10.5% at λ = 1.78611 Å we obtain *a* = 6.443265 Å. This creates conditions for effective high-precision control of chemical composition and strain distribution during growth of heterostructures.

During experimental implementation of the studied case a question arose about criteria to distinguish a single peak from two very close peaks (in Fig. 1 ▸) with neighbouring reflections by comparing their widths. When two peaks overlap we can obtain two different cases: simple overlapping of intensities or new multi-beam reflections caused by dynamical interaction of diffracted waves. The second case corresponds to coincidental diffraction (Chang, 1984 ▸) and is studied in the present paper.

### 3.2. Zn(Mn)Se/GaAs(001) systems   

**Figure 2:**
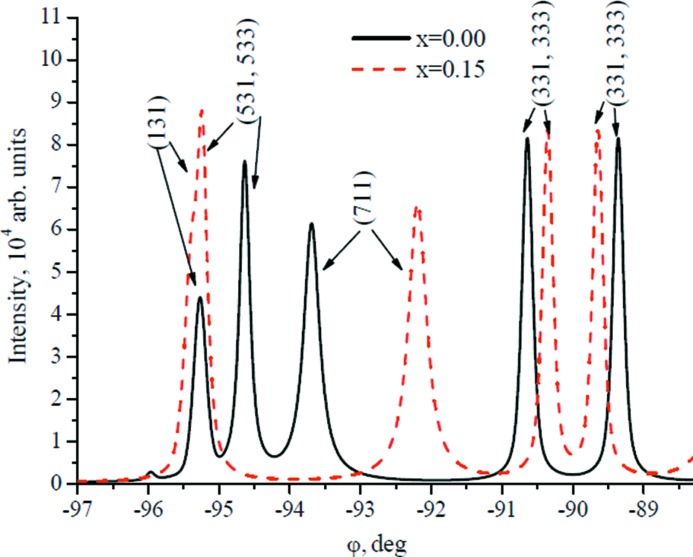
Calculated fragments of diffraction patterns for a two-layered Zn_1−*x*_Mn*_x_*Se system at *x* = 0 and *x* = 0.15, representing substantial changes in not only the angular positions of multi-beam reflections depending on *x* but also their intensities.

**Figure 3:**
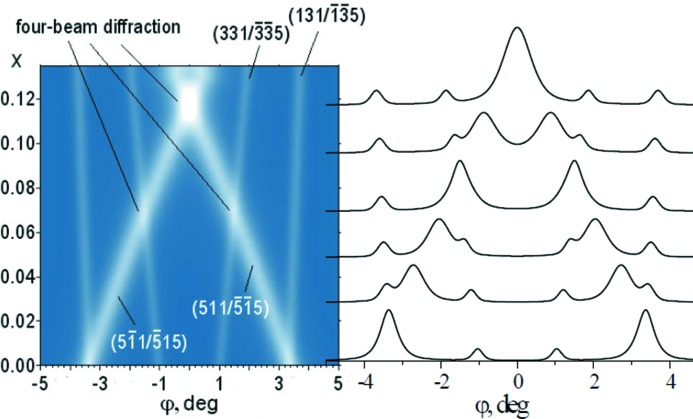
Pattern composed of calculated fragments of diffraction scans with three-beam reflections for Zn_1−*x*_Mn*_x_*Se. Coincidental noncoplanar four-beam diffractions are the result of superposition of three-beam reflections: 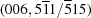 and 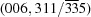, 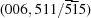 and 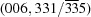, 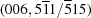 and 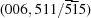 at different values of *x* (λ/*a*).

The MBXRD technique with the coincidental diffraction effect can be used for control of manganese content in multi-layered systems with Zn_1−*x*_Mn*_x_*Se (*x* = 0.05–0.15) layers on a GaAs(001) substrate. The calculation of multi-beam diffraction scans makes it possible to investigate structural disorder at the interfaces of II–VI/III–V heterovalent surfaces, the degree of interdiffusion between chemical elements of Zn(Mn)Se and GaAs layers, and the interface depth of heterovalent mixing for multi-layered structures based on Zn(Mn)Se. Fragments of diffraction scans for crystal structures with Zn_1−*x*_Mn*_x_*Se layers are given in Figs. 2 ▸ and 3 ▸. Reflections from the substrate are disregarded. Fig. 3 ▸ shows three particular cases that represent coincidental noncoplanar four-beam diffraction.

**Figure 4:**
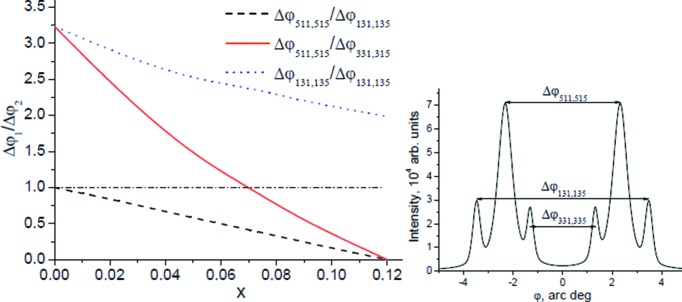
Relative changes of the ratio 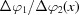 for the three systems of three-beam reflections shown on the right.

In Fig. 4 ▸ the 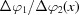 dependences are nonlinear and demonstrate high sensitivity to changes of *x*, thus enabling us to determine the anisotropy and dynamics of the lattice parameter changes.

### 3.3. Lattice strains   

During heteroepitaxial growth, strains (compression or tensile stresses) appear owing to parameter mismatch between two successive layers. As a rule, lattice parameters in the growth direction *a*_⊥_ (normal to the surface) and in the growth plane *a*_||_ are different. This results in tetragonal crystal lattice distortion. Moreover, in separate layer growth the deviation of the *x* value from the expected value can occur. Consequently, this leads to a deviation from Vegard’s law, according to which the lattice parameter (denoted as *a*_0_) should be proportional to *x*.

**Figure 5:**
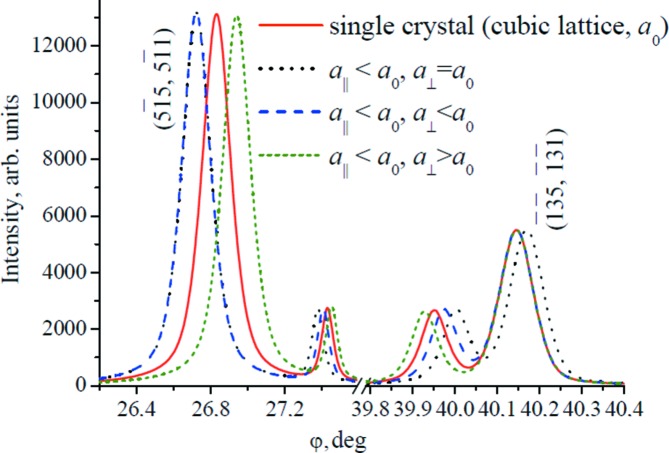
Change in positions of 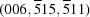 and 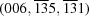 reflections depending on the sign of Δ*a*

The nature and level of tetragonal lattice distortion can be determined from the values of lattice parameters *a*_⊥_ and *a*_||_. In particular, Fig. 5 ▸ shows the dynamics of the changes in the angular positions of multi-beam reflections as a function of the nature of tetragonal lattice distortion by 0.01%. For heterostructures and multi-layered systems the values of *a*_⊥_ and *a*_||_, as well as the values of the relative strains ∊ = Δ*a*/*a*_0_ (Δ*a* = *a*_⊥_ − *a*_||_), enable us to determine how the Mn component is included in the lattice: by substitution of Zn or Se or by formation of point defects of interstitial type.

Thus, examples of simulated scans of X-ray multi-beam diffraction for heterostructures and multi-layered systems demonstrate the opportunities of multi-beam diffraction and can be important both for control of technological processes and for prediction of the electrical and optical properties of such structures, since the majority of properties depend not only on chemical composition but also on the arrangement of atoms in the unit cell.

## 4. Conclusions   

A peculiarity of multi-beam X-ray diffraction patterns is the existence of several systems of related structurally equivalent peaks. Studying the geometry of their angular displacements gives the possibility to determine the changes in the directions and values of the lattice parameters more precisely.

The kinematic approximation of the theory of X-ray scattering in the case of multi-beam X-ray diffraction was used to calculate multi-beam diffraction patterns (Renninger scans) for crystalline Zn_1−*x*_Mn*_x_*Se thin layers and Al*_x_*In_1−*x*_Sb heterostructures. Required conditions (primary reflection and X-ray wavelength) were proposed for each individual layer in the heterostructure to implement three-beam coplanar or four-beam noncoplanar coincidental diffraction. This tool reduces the influence of instrumental errors on the accuracy of the lattice parameter determination.

The possibility to evaluate the tetragonal distortion of the unit cell has been shown for relaxed and nonrelaxed heterostructures using analysis of displacements of different multi-beam reflections that have different behaviours under compressive or stretching strains (the displacement direction depends on the strain sign).
